# Essential Oil from the Aerial Parts of *Artemisia serotina* Bunge (Winter Wormwood) Growing in Kazakhstan—Phytochemical Profile and Bioactivity

**DOI:** 10.3390/molecules30142956

**Published:** 2025-07-14

**Authors:** Arshyn Kadyrbay, Liliya N. Ibragimova, Magdalena Iwan, Agnieszka Ludwiczuk, Anna Biernasiuk, Zuriyadda B. Sakipova, Łukasz Świątek, Kinga Salwa, Agnieszka Korga-Plewko, Karlygash A. Zhaparkulova, Tolkyn S. Bekezhanova, Aleksandra Józefczyk, Jolanta Szymańska, Anna Malm

**Affiliations:** 1 School of Pharmacy, Asfendiarov Kazakh National Medical University, Tole-bi Str. 94, Almaty 000050, Kazakhstan; akadyrbai@vivapharm.kz (A.K.); sakipova.z@kaznmu.kz (Z.B.S.); 2 Pharmacy and Pharmacology Center, Asfendiarov Kazakh National Medical University, Tole-bi Str. 94, Almaty 000050, Kazakhstan; 3 Department of Toxicology, Medical University of Lublin, Chodzki 6 Str., 20-093 Lublin, Poland; magda.iwan@umlub.pl; 4 Department of Pharmacognosy with the Medicinal Plant Garden, Medical University of Lublin, Chodzki 1 Str., 20-093 Lublin, Poland; agnieszka.ludwiczuk@umlub.pl (A.L.); aleksandra.jozefczyk@umlub.pl (A.J.); 5 Department of Pharmaceutical Microbiology, Medical University of Lublin, Chodzki 1 Str., 20-093 Lublin, Poland; anna.biernasiuk@umlub.pl; 6 Department of Virology with Viral Diagnostics Laboratory, Medical University of Lublin, Chodzki 1 Str., 20-093 Lublin, Poland; lukasz.swiatek@umlub.pl (Ł.Ś.); kinga.salwa@umlub.pl (K.S.); 7 Independent Medical Biology Unit, Medical University of Lublin, Jaczewskiego 8b Str., 20-090 Lublin, Poland; agnieszka.korga-plewko@umlub.pl; 8 Department of Biotechnology and General Chemical Engineering, Asfendiarov Kazakh National Medical University, Tole-bi Str. 94, Almaty 000050, Kazakhstan; zhaparkulova.k@kaznmu.kz; 9 Department of Engineering Disciplines and Good Practices, Asfendiarov Kazakh National Medical University, Tole-bi Str. 94, Almaty 000050, Kazakhstan; bekezhanova.t@kaznmu.kz; 10 Department of Comprehensive Paediatric and Adult Dentistry, Medical University of Lublin, Chodzki 6 Str., 20-093 Lublin, Poland; jolanta.szymanska@umlub.pl

**Keywords:** *Artemisia serotina* Bunge, Central Asia, irregular monoterpenes, santolina alcohol, new chemotype, antifungal activity, *Candida albicans*, anticancer activity, breast carcinoma

## Abstract

*Artemisia serotina* Bunge represents one of the endemic *Artemisia* L. species in flora of Central Asia. There is scant information on the phytochemistry and biological activity of this species. The aim of the present study was to analyze the chemical composition of essential oil from *A. serotina* (ASEO) growing in south Kazakhstan, together with the determination of its biological activity. ASEO isolation was carried out by hydrodistillation according to the State Pharmacopoeia of the Republic of Kazakhstan. Analysis of GC/MS data revealed that the most characteristic components of ASEO were irregular monoterpenes from three families: santolinane, artemisane, and lavandulane. The major compound was santolina alcohol (34.6%). Antimicrobial activity was studied against the reference bacterial and fungal strains using the recommended methods, allowing for an estimation of MIC (minimum inhibitory concentration). ASEO was most effective against *Candida albicans* (MIC = 2 mg/mL), exerting fungicidal activity. Thw MIC for bacterial species was higher, i.e., 4–16 mg/mL. Antiviral activity was tested against Coxsackievirus B3 (CVB3) and Human Herpesvirus type 1 (HHV-1) propagated in VERO cells. No antiviral effect against either virus was found at an ASEO concentration of 0.25 mg/mL, but a noticeable decrease in the intensity of HHV-1-related cytopathic effects was observed. Anticancer activity studies included several cancer cell lines. Cytotoxicity, cell cycle, thiol levels, and cell vitality were analyzed. Among the cancer cell lines tested, the breast cancer T47-D cell line exhibited the highest sensitivity to ASEO (IC_50_ = 40.81 ± 4.21 µg/mL at 24 h; IC_50_ = 33.17 ± 2.11 µg/mL at 48 h). The anticancer effect was suggested to be mainly due to the induction of cytostatic effects, accompanied by a disturbance of the intracellular redox balance. The obtained data provide novel information on the unique chemical composition of ASEO from south Kazakhstan, representing a new chemotype. Its bioactivity, including promising antifungal and anticancer properties, was demonstrated for the first time.

## 1. Introduction

The *Artemisia* L. genus, belonging to the Asteraceae family, comprises over 500 species, mainly distributed in the Northern Hemisphere. These plants occur in the wild in North America, Europe, and Asia [1,2,3]. The main center of the species diversity of the *Artemisia* genus is located in Central Asia, covering the territories of Uzbekistan, Tajikistan, Turkmenistan, Kazakhstan, and Kyrgyzstan, as well as parts of Russia, China and Mongolia. In Asia, this genus is represented by about 350 species. In total, 81 species occur in Kazakhstan, including 19 endemic plants [4]. *Artemisia* species are annuals, biennials, and perennials characterized by a wide range of morphological variability. They are shrubs with a characteristic taproot or a partially horizontally arranged rhizome. Their presence is clearly marked in arid and semi-arid steppe areas [1,2,3,4].

Many *Artemisia* plants, like *A. absinthium* L, *A. annua* L., *A. arborescens* (Vaill.) L., *A. campestris* L., *A. cina* O.C. Berg et C.F. Schmidt, *A. dracunculus* L., and *A. vulgaris* L., have been used for centuries in folk medicine, including traditional Asian medicine, for the treatment of several diseases, such as malaria, hepatitis, cancer, inflammation, and infections caused by fungi, bacteria and viruses. This is related to their multiple healing effects: antibacterial, antifungal, antiviral, antipyretic, antimalarial, antitumor, antioxidant, hepatoprotective, anti-ulcer, cholagogic, and decongestant effects [1,2,4,5,6,7]. The main bioactive constituents of *Artemisia* species are terpenoids, flavonoids, phenolic acids (especially caffeoylquinic derivatives), coumarins, sterols, and polyacetylene compounds [5,6].

Essential oils (EOs) from the *Artemisia* genus have been a subject of extensive research. They have been reported to contain mostly monoterpenes. In addition, di-, sesqui-, and triterpenes, as well as sesquiterpene lactones, have been isolated. Several constituents, like 1,8-cineole (eucalyptol), β-pinene, thujones, artemisia ketone, camphor, β-caryophyllene, camphene, and germacrene D, are dominant, depending on the species [4,6,8,9]. The most known secondary metabolite is artemisinin, a sesquiterpene endoperoxide lactone possessing antimalarial activity, widely distributed among several *Artemisia* plants. *A. annua* is a key commercial source of this compound [10]. Moreover, *Artemisia* EOs have been reported to possess a variety of biological activities, including antioxidant, antimicrobial (antibacterial, antifungal, antiviral), insecticidal, repellent, and anticancer properties [6,8,9].

Nurlybekova et al. [4] provided a comprehensive review of *Artemisia* species from Central Asia, including *A. serotina* Bunge (winter wormwood), in terms of botanical, phytochemical, and pharmacological aspects, together with their traditional uses. This plant represents an endemic species growing in the territories of Kazakhstan and Uzbekistan. It is a perennial shrub and is 40–80 cm high. This plant grows in different locations, like desert zones, saline clayey and sandy loamy soils, river terraces, dry soils, and gravel–clay slopes of foothills. It also occurs as a weed on pastures, fallow lands, abandoned arable lands, and near roads [4]. *A. serotina* is not a sufficiently explored species in terms of its ethnopharmacological aspects. There are only a few publications on the chemical composition of EO isolated from the aerial part of *A. serotina* (further called ASEO) from plants collected in Kazakhstan and Uzbekistan [4,11,12]. The first report about ASEO isolated from Kazakh plant species, published in 1959, showed a domination of α-thujone, followed by 1,8-cineole, carvone, and camphor [11]. Later data published in 2017 on ASEO isolated from plants growing in Uzbekistan revealed chrysanthenon, 1,8-cineole, and filifolid A as the main components [11]. Recently, GC/MS analysis of the hexane fraction obtained from the hydroethanolic extract (70% *v*/*v*) of the underground parts of Kazakh *A. serotina* plants revealed the presence of 23 liposoluble compounds, mostly chrysanthenone, 1,8-cineole, ascaridol, and 3-(5-methyl-5-vinyltetrahydrofuran-2-yl) butan-2-ol [13].

The aim of this study was to analyze the chemical composition of ASEO isolated from the aerial parts of plants derived from the native flora of south Kazakhstan in comparison to the compositional data on Kazakh and Uzbek species available in the literature. Moreover, the antibacterial, antifungal, antiviral, and anticancer activities of this EO were assessed for the first time to fill the gap in its bioactivity data.

## 2. Results

### 2.1. Chemical Composition

ASEO was obtained from the aerial part of plants growing at the foothills of the Transili Alatau in south Kazakhstan. The hydrodistillation method was used, with a yield of 1%.

**Figure 1:**
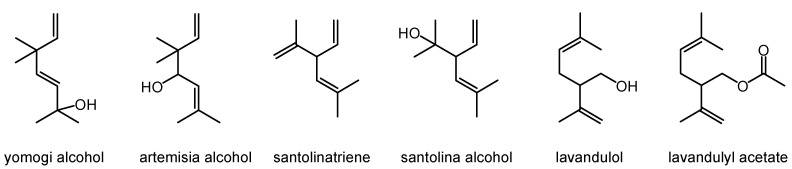
Irregular monoterpenes in ASEO.

The data presented in Table 1 show that, in total, 23 compounds were identified in ASEO, constituting 89.5% of its composition. The most characteristic components of ASEO were irregular monoterpenes belonging to three families: santolinane, artemisane, and lavandulane. The major compound of the investigated EO was santolina alcohol. The relative percentage of this compound was 34.6%. Other compounds from the group of irregular monoterpenes were santolinatriene, yomogi alcohol, artemisia alcohol, lavandulol, and lavandulyl acetate. The chemical structures of the irregular monoterpenes characteristic of this ASEO are presented in Figure 1.

Besides irregular monoterpenes, common (regular) monoterpenes were also identified in ASEO. These were camphor (7.6%), trans-p-menth-2-en-1-ol (3.0%), camphene, borneol, terpinen-4-ol, and a-terpineol. The presence of sesquiterpenes (bicyclogermacrene, germacrene D, sptathulenol, and globulol) was also confirmed. Among the EO components, there were also four compounds that were not identified. These were compounds with retention indices of 1030, 1159, 1164, and 1249. They constituted 38.5% of all ASEO components. Mass spectra suggest that these compounds could belong to irregular monoterpenes (Figure S1 in Supplementary Materials).

### 2.2. Antibacterial and Antifungal Activity

The data presented in Table 2 indicate that ASEO showed potential activity against the reference Gram-positive and Gram-negative bacterial strains, with MICs ranging from 4 to 16 mg/mL and MBCs from 8 to >16 mg/mL. The calculated MBCs/MICs were within the range 1–2, resulting in a bactericidal effect of ASEO. *Micrococcus luteus* ATCC 10240, representing Gram-positive bacteria, was the most sensitive to ASEO, with MIC = 4 mg/mL. The remaining strains, belonging to Gram-positive bacteria, namely *Staphylococcus aureus* ATCC 29213, *Staphylococcus aureus* ATCC 43300, *Staphylococcus epidermidis* ATCC 12228, *Enterococcus faecalis* ATCC 29212, *Bacillus subtilis* ATCC 6633, and *Bacillus cereus* ATCC 10876, were less susceptible to this essential oil (MIC = 8 to 16 mg/mL). A similar sensitivity to ASEO was observed for Gram-negative bacteria from Enterobacterales, i.e., *Escherichia coli* ATCC 25922 (MIC = 8 mg/mL) and *Salmonella* Typhimurium ATCC 14028 (MIC = 16 mg/mL), as well as from non-fermenting rods, i.e., *Pseudomonas aeruginosa* ATCC 9027 (MIC = 8 mg/mL).

According to data from Table 2, *Candida albicans* ATCC 10231, belonging to fungi (yeasts), was more sensitive to ASEO in comparison to the bacterial species studied. ASEO showed antifungal activity with MIC = 2 mg/mL and MFC = 4 mg/mL. Consequently, a MFC/MIC ratio of 2 indicated its fungicidal effect.

### 2.3. Antiviral Activity

Before the assessment of the antiviral activity of ASEO, a non-cytotoxic concentration of this essential oil was selected for VERO cells used for Human Herpesvirus type 1 (HHV-1) and Coxsackievirus B3 (CBV3) multiplication. Since ASEO at 500 µg/mL decreased the viability of these cells after 72 h incubation by approx. 35%, 250 µg/mL was used as the maximum non-toxic concentration.

**Figure 2:**
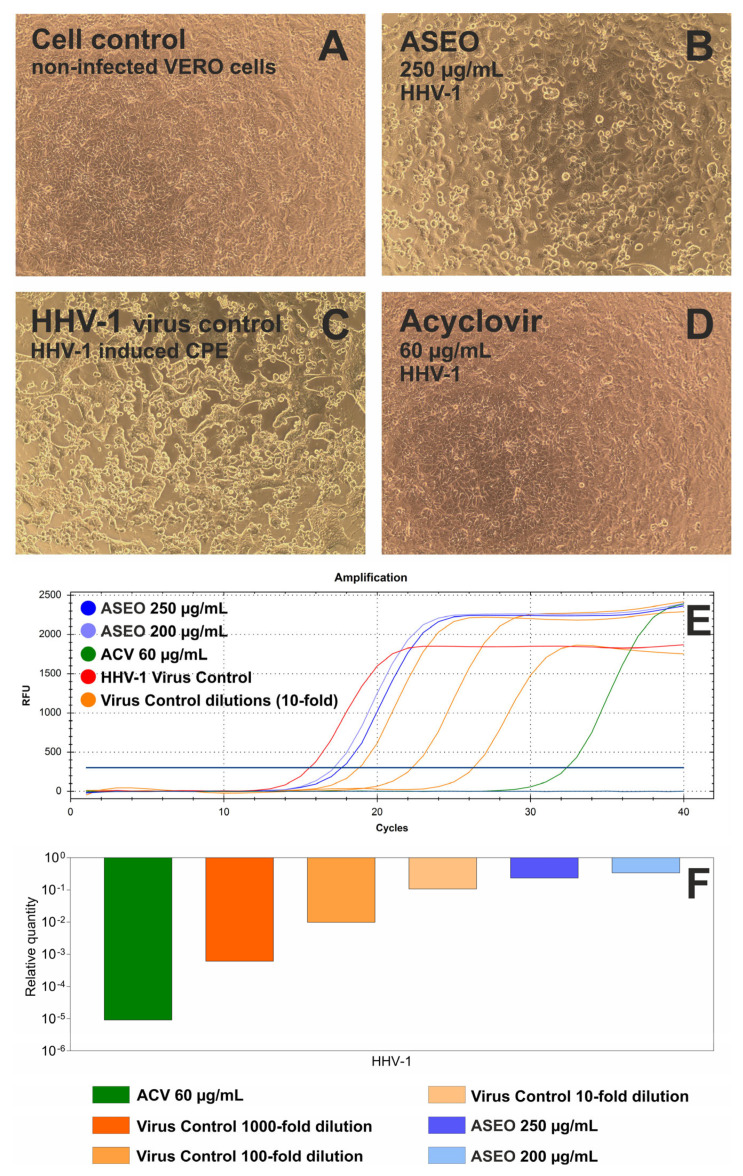
Antiviral activity of ASEO against (**A**) HHV-1-non-infected VERO cells, cell control; (**B**) influence of ASEO 250 µg/mL on HHV-1-infected VERO cells; (**C**) HHV-1-induced cytopathic effect, HHV-1 virus control; (**D**) influence of acyclovir (ACV) 60 µg/mL on HHV-1-infected VERO cells; (**E**) qPCR amplification of HHV-1 DNA; (**F**) reduction in HHV-1 viral load.

The typical CPE induced by HHV-1 in VERO cells is presented in Figure 2A. The incubation of HHV-1-infected VERO cells with ASEO at a concentration of 250 µg/mL (Figure 2B) noticeably decreased the intensity of CPE when compared with the virus control (Figure 2C). The qPCR analysis (Figure 2E) revealed that ASEO at the same concentration (250 µg/mL) reduced the HHV-1 viral load (Figure 2F) by 0.63 log. Acyclovir, which is a reference antiviral drug against HHV-1, used at 60 µg/mL prevented the development of CPE in HHV-1-infected VERO cells (Figure 2D) and reduced HHV-1 viral load by 5.05 log (Figure 2F).

Data on the activity of ASEO against CVB3 are included in Figure S2 in the Supplementary Materials. The typical CPE induced by CVB3 in VERO cells (CVB3 virus control) is presented in Figure S2A. ASEO treatment did not influence the formation of CPE (Figure S2B). In contrast, a broad-spectrum antiviral drug, ribavirin (RBV), managed to dose-dependently decrease the CPE, as shown in Figure S2C,D. According to RT-qPCR results (Figure S2E,F), ASEO at 250 µg/mL reduced the CVB3 viral load by 0.63 log, while RBV at 500 and 250 µg/mL reduced the viral load by 2.03 and 1.35 log, respectively.

### 2.4. Anticancer Activity

The data presented in Table 3 suggest that ASEO exhibited selective cytotoxicity and was more effective against specific cancer cell lines. Among the tested cell lines, the breast cancer T47-D cell line exhibited the highest sensitivity, with IC_50_ values of 40.81 ± 4.21 µg/mL at 24 h and 33.17 ± 2.11 µg/mL at 48 h incubation, indicating an increase in potency over time. Malignant melanoma G-361 cells showed moderate susceptibility, with IC_50_ values decreasing from 273.22 ± 2.58 µg/mL at 24 h to 252.51 ± 3.45 µg/mL at 48 h, suggesting slightly enhanced cytotoxicity with prolonged exposure. A comparable trend was also observed in the NCI-H1563 lung cancer cell line. The values of IC_50_ at 24 h for FaDu (393.88 ± 18.19) and RKO (346.15 ± 29.35) cancer cell lines were higher. However, an almost twofold decrease in IC_50_ values for these cell lines was observed at 72 h, at 230.58 ± 19.01 and 161.95 ± 6.15, respectively.

The remaining cancer cell lines exhibited an insensitivity to the tested ASEO, with IC_50_ values nearing or exceeding 500 µg/mL, suggesting a minimal cytotoxic impact. A similar degree of insensitivity was noted in the normal cell lines MCF-10, HUVEC, and BJ, suggesting that ASEO did not exhibit cytotoxic effects on healthy cells.

**Figure 3:**
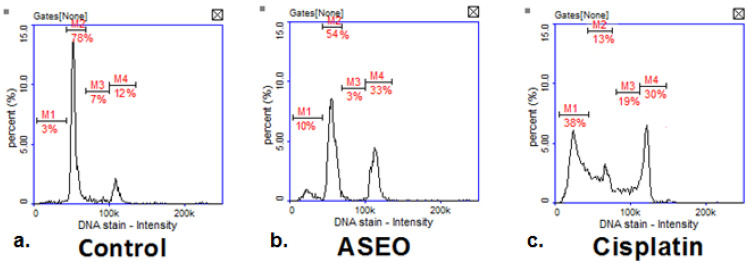
Cell cycle analysis in T47D breast cancer cells treated with ASEO at a concentration of IC_50_ = 33.17 µg/mL. Cisplatin was used as positive control.

The cell cycle analysis in the breast cancer T47-D cell line using image cytometry demonstrated noticeable alterations in the populations of cells treated with ASEO (at IC_50_ concentration) compared to the control group (Figure 3a,b). In the untreated control, cells exhibited a typical cell cycle distribution, 78% in the G0/G1 phase, 7% in the S phase, and 12% in the G2/M phase, with minimal DNA fragmentation (sub-G1, 3%), indicating normal cell proliferation and negligible apoptosis (Figure 3a). Treatment with ASEO resulted in a significant G2/M phase arrest (33%), alongside reductions in the G0/G1 (54%) and S phases (3%). DNA fragmentation increased to 10%, suggesting a primarily cytostatic effect with some induction of apoptosis (Figure 3b). As the positive control, cisplatin produced pronounced effects, including extensive DNA fragmentation (sub-G1, 38%) and significant disruption of the cell cycle, with 13% of cells in G0/G1, 19% in S, and 30% in G2/M (Figure 3c). These results suggest that the tested ASEO induced mainly cytostatic effects with moderate apoptotic activity at its IC_50_ value.

**Figure 4:**
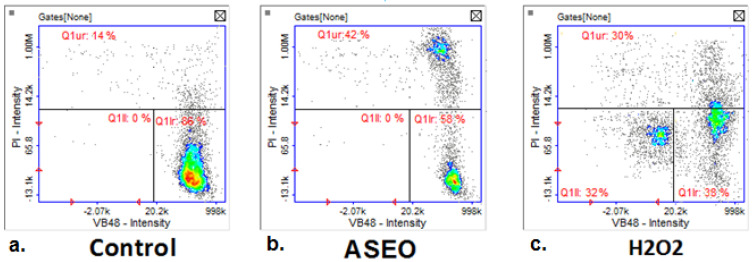
Analysis of cell vitality and thiol levels in the T47D breast cancer cell line treated with ASEO at a concentration of IC_50_ = 33.17 µg/mL. Hydrogen peroxide (H_2_O_2_) was used as a positive control for oxidative stress.

In the untreated control of the breast cancer T47-D cell line, the majority of the cells (86%) were viable with high thiol levels, as shown by the strong VB-48™ fluorescence (Q1lr quadrant) (Figure 4a). Only a small proportion (14%) displayed signs of stress or low vitality (Q1ur quadrant), with minimal cell death detected (negligible PI staining in other quadrants). Cells treated with ASEO showed a significant shift in population dynamics (Figure 4b). Viable cells with high thiol levels decreased to 58% (Q1lr quadrant), indicating reduced vitality. A notable increase in the population with low thiol levels (Q1ur quadrant, 42%) reflects stress or apoptotic changes induced by ASEO. Importantly, no major increase in necrotic cells was observed (negligible PI-positive staining), suggesting that ASEO predominantly affects intracellular redox balance without directly compromising membrane integrity. Treatment with hydrogen peroxide resulted in substantial oxidative stress (Figure 4c). A marked increase in cells with low thiol levels and compromised vitality was observed, with 30% of cells in Q1ur and 32% of cells in Q1ll. Additionally, only 38% of cells retained high thiol levels (Q1Ir), reflecting a significant reduction in vitality. The population in Q1ur also indicates necrosis or severe membrane damage induced by H_2_O_2_.

## 3. Discussion

The genus *Artemisia* is characterized by great phytochemical variability. The composition of EOs from *Artemisia* species, as well as their quality and yield, varies depending on several factors, including the plant part, chemotype, subspecies, harvesting season, age of the plant, geographic location, altitude, extraction techniques, solvent, and timing. The EO composition, even within the same species, may vary widely, representing different chemotypes [1,6,7,9].

The phytochemical composition of ASEO originating from Uzbekistan and Kazakhstan was previously screened. These studies revealed great differences in its chemical constituents, depending on the region. EO from Kazakhstan showed domination of α-thujone (53.9%), while that from Uzbekistan contained mostly chrysantenon (13.0%), 1,8-cineole (10.08%), and filifolid A (8.62%) [4,11,12]. The chemical composition of the ASEO described in this paper is unique among the essential oils of the *Artemisia* plant [1,7,9] and different from that in previous studies concerning this EO [11,12]. It is characterized by the presence of irregular monoterpenes from three families, santolinane, artemisane, and lavandulane, and santolina alcohol (34.6%) as a main compound, representing a new chemotype of ASEO. It is worth noting that santolina alcohol (40.8%) and camphor (32.4%) were the major volatiles of EO from *Artemisia sieberi* Besser growing in Iran, constituting a distinct chemotype—santolina alcohol/camphor [14]. Four other chemotypes were also defined in this region, namely (i) camphor/α-thujone, (ii) camphor/1,8-cineole, (iii) α-thujone/β-thujone/camphor, and (iv) α-thujone/borneol/β-thujone, confirming the intraspecies diversity of *A. sieberi*. The data on ASEO composition presented here, together with data in the literature [14], suggest a similar chemotype variation also within *A. serotina* species. Moreover, santolina alcohol, together with 1,8-cineole, camphor, and spathulenol, were the major components of *Artemisia turcomanica* Gand from Iran [15]. Other data indicate that santolina alcohol together with yomogi alcohol and artemisyl acetate were the most characteristic components of EO obtained from *Artemisia ageratum* from Corsica. However, the major compound of this oil was 1,8-cineole [16]. In addition, santolina alcohol was also found to be present in other *Artemisia* species, including *A. kermanensis* and *A. vestita* [1,6,9].

EOs from several *Artemisia* species, like *A. absinthium*, *A. annua*, *A. gmellini*, *A. Judaica,* and *A. vulgaris*, have been explored for their antibacterial and antifungal properties against several human pathogens by using the disk diffusion method for screening purposes and agar or broth dilution method allowing for MIC estimation [7,17,18]. Antimicrobial effects have been ascribed to the presence of various classes of EO compounds, including monoterpenes, sesquiterpenes, and phenylpropanoids [18,19].

This is the first report on the antibacterial activity of ASEO and its bactericidal effect. The MIC values of this EO for reference strains belonging to both Gram-positive (4–16 mg/mL) and Gram-negative bacteria (8–16 mg/mL) were relatively high. Antibacterial activity was also reported for the EO from *A. sieberi* belonging to the santolina alcohol/camphor chemotype. This EO possessed bactericidal properties as well. Higher MIC values was estimated for *S. aureus* (12.5 mg/mL), as well as for *E. coli* and *P. aeruginosa* (25 mg/mL) [14], as compared to those of ASEO. The screening of EO from *A. turcomanica* by the disk diffusion method also revealed its broad-spectrum antibacterial activity [15]. It is worth noting that *A. annua* represents the most studied species in terms of antibacterial properties. These properties have been assigned to the presence of the main compounds of its EO, mostly artemisia ketone. Due to great differences in the content of this compound (0.1–68.5%), the antibacterial potential of *A. annua* EOs has been reported to differ significantly. This was revealed by a MIC of 0.008–32 mg/mL for Gram-positive bacteria and of 0.03–64 mg/mL for Gram-negative bacteria [20]. Ma et al. [21] found that the MIC values of *A. annua* essential oil containing 31.26% artemisia ketone for both *S. aureus* and *E. coli* were 0.02 mg/mL.

The antifungal activity of ASEO is presented in this paper for the first time. ASEO inhibited the growth of the reference strain of *C. albicans* with MIC = 2 mg/mL, showing a fungicidal effect. According to data in the literature [14], EO from *A. sieberi* of the santolina alcohol/camphor chemotype exerted a higher MIC = 12.5 mg/mL for *C. albicans*. Moreover, EOs from various *Artemisia* species, like *A. annua L*., *A. dracunculus* L., *A. abrotanum* L., *A. judaica* L., and *A. vulgaris* L. were found to possess activity against both reference and clinical strains of *Candida* spp., including not only *C. albicans* but also *C. glabrata*, *C. parapsilosis*, and *C. dublinienisis* [7,20,22]. For example, the MIC of *A. annua* EO for *C. albicans* was reported to be 2–20 mg/mL. The antifungal activity of this EO has been assigned to α-terpineol [21]. It is worth noting that EOs can be regarded as an alternative treatment for superficial fungal infections, including candidiasis [23]. In the light of the presented data, ASEO can be considered a promising antifungal agent for topical application, e.g., in oral candidiasis [24].

According to the literature data, EOs from several *Artemisia* species were studied for their antiviral properties [25]. The evaluation of antiviral activity of the ASEO presented here was based on studies using two human viruses, CVB3 and HHV-1, representing RNA and DNA viruses, respectively. HHV-1 is also known as Herpes Simplex Virus type 1 (HSV-1) [26]. Despite a noticeable decrease in the intensity of the HHV-1-induced cytopathic effect in ASEO-treated virus-infected VERO cells, no significant antiviral activity of ASEO was observed at the maximum non-toxic concentration (0.25 mg/mL) which could be used in the experiments. Importantly, only a 0.63 log reduction in the HHV-1 and CBV3 viral loads was observed in the ASEO-treated virus-infected VERO cells. It should be noted that a successful antiviral drug candidate is expected to reduce the viral load or infectious titer by more than 2 log. This is why the decision was made not to pursue further antiviral studies, which could have identified a mechanistic basis, such as pinpointing which step of the viral replication was blocked. Such studies are validated when screening assays show a significant inhibition of viral replication. In turn, Saddi et al. [27] found that EO obtained from *A. arborescens*, endemic species of the Mediterranean region, possessed anti-herpesviral activity. The plaque reduction assay revealed dose-dependent virucidal activity at low IC_50_ (50% inhibitory concentration) values of 2.4 and 4.1 μg/mL for HSV-1 and HSV-2, respectively. However, there were no antiviral effects in virus attachment and penetration assays or during post-attachment experiments. The activity of EO from *A. arborescens* was also confirmed by Lai et al. [28], together with a proposal of a skin delivery system based on solid lipid nanoparticles. This EO was reported to contain predominantly chamazulene, camphor, or trans-thujone, depending on the country [29].

*Artemisia* species can be regarded as a rich source of bioactive compounds with antitumor activity, including terpenoids [30]. Cytotoxic and anticancer properties have been described for EOs of several *Artemisia* species, like *A. arborescens,* containing a high content of chamazulene (52%) [18], and *A. campestris* L., with the identified key compounds including linalyl acetate (2.92%), geranyl acetate (2.45%), and eucalyptol (1.38%) [31]. Moreover, EO obtained from *A. sieberi* growing in Saudi Arabia, a medicinal herb traditionally used in the Middle East to treat cancer, was studied for cytotoxicity against the HCT116, HepG2, A549, and MCF-7 cancer cell lines. This EO exerted a cytotoxic effect against them in a dose-dependent manner, with IC_50_ values of 53.1 μg/mL, 56.6 μg/mL, 60.7 μg/mL, and 38.7 μg/mL, respectively. The anticancer effect against MCF-7 cells resulted from a complex mechanism involving S-phase cell cycle arrest, caspase-independent apoptosis, and downregulation of the ERK (extracellular signal-regulated kinases) pathway [32]. The chemical composition of this EO was quite different than that reported elsewhere for *A. sieberi* growing in Iran [14]. The major compound was found to be cis-crysanthenyl acetate (48.56%), followed by davanone (10.58%), 1,8-cineole (6.81%), and caryophyllene diepoxide (5.34%) [32]. Among the cancer cell lines tested, the breast cancer T47-D cell line exhibited the highest sensitivity to ASEO (IC_50_ = 40.81 ± 4.21 µg/mL at 24 h; IC_50_ = 33.17 ± 2.11 µg/mL at 48 h). The anticancer effect was suggested to be mainly due to the induction of cytostatic effects, accompanied by a disturbance of intracellular redox balance. Herein, the anticancer activity of ASEO was studied against several cancer cell lines, including two cell lines of breast adenocarcinoma, MCF-7 and T47-D. Among them, the breast cancer T47-D cell line exhibited the highest sensitivity to ASEO (IC_50_ = 40.81 ± 4.21 µg/mL at 24 h, IC_50_ =33.17 ± 2.11 µg/mL at 48 h). The anticancer mechanism of ASEO was suggested to be mainly due to the induction of cytostatic effects, with moderate apoptotic activity, revealed as a significant G2/M phase arrest together with a reduction in the G0/G1 and S phases. A disturbance of intracellular redox balance without a direct effect on membrane integrity was also observed. Elsewhere, the mechanisms underlying the antitumor effect of *Artemisia* species have been suggested to be due to the induction of apoptosis, cell cycle arrest, and/or the induction of reactive oxygen species production [30]. Moreover, several medicinal plants, including those from the Asteraceae family (*A. monsperma* Delile), have been considered to contain bioactive compounds with activity against breast cancer, based on preclinical and clinical data [33].

Due to the great intraspecies diversity of *Artemisia* plants [1,6,7,9], there is a need to correlate its bioactivity to a given chemotype in terms of its potential health applications. Irregular monoterpenes are characteristic of the *Artemisia* genus and the entire Asteraceae family. It is worth noting that the ASEO isolated from the aerial part of native plants growing in south Kazakhstan was found to represent a new chemotype containing irregular monoterpens belonging to three families: santolinane, artemisane, and lavandulane. Santolina alcohol was the main compound (34.6%). The bioactivity data of this EO revealed its promising antifungal activity against *Candida albicans,* followed by further formulation studies allowing for the development of novel topical preparations, including those containing ASEO and known antifungals. Moreover, the cytostatic properties of ASEO against T47D breast cancer can be regarded as a basis for further in-depth research. A limitation of the present study is the relatively high percentage of unidentified compounds (38.5%), suggested to belong to irregular monoterpens. Therefore, there is a need for the further isolation of these compounds, the determination of their structure, and the screening of their bioactivity [14,18,20,21,31].

## 4. Materials and Methods

### 4.1. Plant Material

The collection of wild-growing plant materials of *Artemisia serotina* Bunge was carried out in accordance with the requirements of GACP and standard operating procedures of the pharmaceutical enterprise Fitoleum LLP (Esik, Kazakhstan) in the Almaty region, on the foothill plain of the western end of the Transili Alatau (N 43°20′32.0″ E 075°32′47.0″) in the period from mid-August to mid-September 2022. Species identification was carried out at the Republican State Enterprise on the Right of Economic Management “Institute of Botany and Phytointroduction” of the Republic of Kazakhstan. The above-ground part of the plant was cut off at a length of 15–25 cm from the top, using a manual method of collection, in the morning from 7:00 to 10:00.

### 4.2. Method of Quantitative Isolation of Essential Oil from Plant Material

The isolation of essential oil from plant material was carried out by hydrodistillation according to the State Pharmacopoeia of the Republic of Kazakhstan I, Vol. 1, 2.8.12 (Method 1) [34]. Essential oils were determined by the Ginzberg method: about 50 g (accurately weighed) of crushed raw material was placed in a round-bottomed flask with a capacity of 1000 mL; 300 mL of hot purified water was added, a reflux condenser with a graduated nozzle was attached and heated for 2 h, and the essential oils accumulated as they were distilled with steam in a graduated test tube. The volume of oil was measured after the device had cooled to room temperature.

### 4.3. GC/MS Analysis

Analysis was performed with a Shimadzu GC-2010 Plus GC instrument coupled to a Shimadzu QP2010 Ultra mass spectrometer (Shimadzu Europa GmbH, Duisburg, Germany). Compounds were separated on a fused silica capillary column ZB-5 MS (30 m, 0.25 mm i.d.) with a film thickness of 0.25 mm (Phenomenex, Torrance, CA, USA). An oven temperature program was initiated at 50 °C and held for 3 min and then increased to 250 °C at a rate of 5 °C/min and held for 15 min. The spectrometers were operated in electron impact mode; the scan range was 40–500 amu, the ionization energy was 70 eV, and the scan rate was 0.20 s per scan. The injector, interface, and ion source were kept at 250, 250, and 220 °C, respectively. Split injection was conducted with a split ratio of 1:20 and helium was used as carrier gas of 1.0 mL/min flow rate. The retention indices were determined in relation to a homologous series of n-alkanes (C8–C24) under the same operating conditions. Compounds were identified using computer-assisted spectral libraries (MassFinder 2.1; MassFinder 4.2; NIST 2011).

### 4.4. Antimicrobial Activity Assay

ASEO was investigated in vitro for antibacterial and antifungal activities. The reference strains of microorganisms from American Type Culture Collection (ATCC) (Manassas, VA, USA) were included. The representatives of Gram-positive bacteria were *Staphylococcus aureus* ATCC 29213, *Staphylococcus aureus* ATCC 43300, *Staphylococcus epidermidis* ATCC 12228, *Enterococcus faecalis* ATCC 29212, *Micrococcus luteus* ATCC 10240, *Bacillus subtilis* ATCC 6633, and *Bacillus cereus* ATCC 10876, while those of Gram-negative bacteria were *Escherichia coli* ATCC 25922, *Salmonella* Typhimurium ATCC 14028, and *Pseudomonas aeruginosa* ATCC 9027. Moreover, a fungal strain belonging to yeasts, *Candida albicans* ATCC 10231, was included.

The antimicrobial testing of ASEO was performed using the microdilution broth method [35] in accordance with the guidelines of the European Committee on Antimicrobial Susceptibility Testing (EUCAST) [36] and the Clinical and Laboratory Standards Institute (CLSI) [37]. All microbial cultures used were first subcultured on nutrient agar (for bacteria) or Sabouraud agar (for fungi) (BioMaxima S.A., Lublin, Poland) and incubated at 35 °C for 18–24 h. Microbial suspensions were prepared in sterile saline (0.85% NaCl) with an optical density of 0.5 McFarland standard scale corresponding to defined numbers of CFUs (Colony-Forming Units), at 1.5 × 10^8^ CFU/mL for bacteria and 5 × 10^6^ CFU/mL for yeasts. ASEO was first dissolved in dimethyl sulfoxide (DMSO) to obtain a concentration of 100 mg/mL. The minimal inhibitory concentrations (MICs) of ASEO were evaluated by the microdilution broth method in 96-well polystyrene microplates. In this study, two-fold dilutions of this essential oil was prepared in Mueller–Hinton agar (BioMaxima S.A., Lublin, Poland) for bacteria and Mueller–Hinton agar with 2% glucose for fungi (BioMaxima S.A., Lublin, Poland). The final concentrations of ASEO (diluted in broth) ranged from 0.008 to 16 mg/mL. Next, bacterial or fungal suspensions were introduced into each well of the microplate to obtain a final density of 1.5 × 10^6^ CFU/mL for bacteria and 5 × 10^4^ CFU/mL for yeasts. After 18–24 h incubation at 35 °C, the MIC value was assessed using a BioTek spectrophotometer (Biokom, Janki, Poland) as the minimal concentration of ASEO that showed complete microbial growth inhibition. The inhibition of bacterial and fungal growth was assessed by comparison with control cultures in media tested without essential oil. Ciprofloxacin or vancomycin (antibacterial drugs) and nystatin (antifungal drug) purchased from Sigma-Aldrich Chemicals, St. Louis, MO, USA, were used as reference substances. Appropriate DMSO, sterile, and growth controls were prepared. Media with and without ASEO was used as controls. Subsequently, the minimal bactericidal concentration (MBC) or minimal fungicidal concentration (MFC) values of ASEO were determined by transferring the cultures from each MIC determination well to the appropriate solid medium. After incubation, the lowest concentrations of ASEO with no visible bacterial or fungal growth were evaluated as MBC or MFC. Moreover, the MBC/MIC or MFC/MIC ratios were calculated in order to determine the bactericidal/fungicidal (MBC/MIC ≤ 4, MFC/MIC ≤ 4) or bacteriostatic/fungistatic (MBC/MIC > 4, MFC/MIC > 4) effect of the tested EO [38].

### 4.5. Determination of Antiviral Activity

#### 4.5.1. Cell Maintenance and Cytotoxicity Testing

The cytotoxicity of ASEO was evaluated for VERO (ATCC: CCL-81; monkey kidney) cells. Dulbecco’s Modified Eagle’s Medium (DMEM; Corning, Tewksbury, MA, USA) was used. The cell medium was supplemented with antibiotics (Penicillin–Streptomycin Solution, 100× concentrate, Corning) and fetal bovine serum (FBS; Corning). Phosphate-buffered saline (PBS) and trypsin were purchased from Corning. 3-(4,5-dimethylthiazol-2-yl)-2,5-diphenyltetrazolium bromide was purchased from Sigma-Aldrich (St. Louis, MO, USA). ASEO was dissolved (50 mg/mL) in DMSO (cell-culture grade purity, PanReac Applichem, Darmstadt, Germany) to obtain the stock solution for cytotoxicity evaluation and antiviral assays.

The microculture tetrazolium assay (MTT) was used to evaluate the cytotoxicity of ASEO towards VERO cells in order to select the non-cytotoxic concentrations for further use in the antiviral assay. The cytotoxicity testing was performed using microculture tetrazolium assay as was previously described [39]. Briefly, monolayers of selected cells in 96-well plates were treated with serial dilutions of ASEO stock solution in cell media for 24 or 72 h. Afterwards, cell media were removed, and after washing with PBS, the MTT-supplemented medium was added and incubated for 3 h. Subsequently, the formazan product was dissolved, and after overnight incubation, the Synergy H1 Multi-Mode Microplate Reader (BioTek Instruments, Inc. Winooski, VT, USA) was used to measure the absorbance (540 and 620 nm). The absorbance data was analyzed using GraphPad Prism (version 10.2.0), and the CC_50_ values (50% cytotoxic concentration) were calculated from dose–response curves (non-linear regression).

#### 4.5.2. Antiviral Assays

Antiviral activity was tested against Human Coxsackievirus B3 (CVB3; ATCC, VR-30) and Human Herpesvirus type 1 (HHV-1, ATCC, VR-260). Both viruses were propagated in VERO cells. The experiments were performed following a previously published protocol [40]. The VERO cells were seeded into a 48-well plate and incubated overnight. Afterwards, the cells were infected with CVB3 or HHV-1 in 100-fold CCID_50_/mL (CCID_50_—50% cell culture infectious dose). At least two wells per plate were left non-infected (cell control). After 1 h of incubation, the virus-infected monolayers were washed with PBS to remove unabsorbed virions, ASEO in non-cytotoxic concentrations was added, and incubation continued until CPE (cytopathic effect) was observed in the virus control (VC, virus-infected, non-treated cells). Subsequently, an inverted microscope (CKX41, Olympus Corporation, Tokyo, Japan) equipped with a camera (Moticam 3+, Motic, Hong Kong) was used to observe the plates and document (Motic Images Plus 2.0, Motic) the results. Finally, the plates were thrice frozen at −76 °C and thawed, and samples were collected to evaluate the viral load. Ribavirin (500 µg/mL) and acyclovir (60 µg/mL) were used as antiviral substances against CVB3 and HHV-1, respectively.

Samples collected from anti-CVB3 assays were subjected to RNA isolation (QIAamp Viral RNA Mini Kit, Cat.: 52,904 QIAGEN GmbH, Hilden, Germany), while DNA was isolated (QIAamp DNA Mini Kit, Cat.: 51,304 QIAGEN GmbH) from samples collected during anti-HHV-1 experiments. The RNA isolates were subjected to one-step RT-qPCR (reverse-transcription quantitative polymerase chain reaction) amplification using an iTaq Universal SYBR Green One-Step Kit (Cat.: 1725150, Bio-Rad Laboratories, Life Science Group, Hercules, CA, USA) and enterovirus-specific primers (entrinR (5′-GAAACACGGACACCCAAAGTA-3′) and entrinF (5′-CGGCCCCTGAATGCGGCTAA-3′)) on a CFX96 thermal cycler (Bio-Rad Laboratories). The RT-qPCR amplification parameters were as follows: reverse-transcription reaction (50 °C, 10 min), polymerase activation (95 °C, 1 min), cycling (40 repeats: denaturation (95 °C, 10 s), annealing and synthesis (65 °C, 30 s), fluorescence acquisition), and melting curve analysis (65–95 °C, 0.5 °C increment/5 s). The DNA isolates from anti-HHV-1 assays were subjected to qPCR amplification with SsoAdvanced Universal SYBR Green Supermix (Bio-Rad Laboratories) and primers (UL54F–5′CGCCAAGAAAATTTCATCGAG 3′, UL54R–5′ ACATCTTGCACCACGCCAG 3′) on the CFX96 thermal cycler. The qPCR amplification conditions were as follows: polymerase activation (98 °C, 3 min); cycling (40 repeats: DNA denaturation (95 °C, 10 s), annealing and synthesis (60 °C, 30 s), fluorescence acquisition); melting curve analysis (65–95 °C). The CVB3 and HHV-1 viral loads in the ASEO-treated samples were evaluated in relation to VC using the relative quantity (ΔCq) method on CFX Manager™ Dx Software (Bio-Rad Laboratories). The sensitivity of RT-qPCR and qPCR was evaluated by analyzing dilutions (10, 100, and 1000-fold) of virus RNA isolate (CVB3) or DNA isolate (HHV-1).

### 4.6. Determination of Anticancer Activity

#### 4.6.1. Cell Culturing and Treatment

The studies were performed on a range of cancer cell lines, including melanoma (G-361), cervical adenocarcinoma (HeLa), pancreatic carcinoma (PANC-1), breast adenocarcinoma (MCF-7 and T47-D), and lung carcinoma (A-549, NCI-H1563, NCI-H2170), as well as hepatocellular carcinoma (Hep-G2) and prostate cancer cell lines (PC-3, DU145, and LNCaP). Cell lines derived from cancers of the colon (RKO; ATCC: CRL-2577, human colon cancer), hypopharynx (FaDu; ATCC: HTB-43, human hypopharyngeal squamous-cell carcinoma), and stomach (AGS; ATCC: CRL-1739, human gastric adenocarcinoma) were also included. The studies also included normal cell lines to assess the potential toxicity of the tested treatments. Specifically, normal breast epithelial cells (MCF-10A), endothelial cells (HUV-EC-C), and normal foreskin fibroblasts (BJ) were used in the analysis. All cell lines used in the studies, including cancer and normal lines, were purchased from the American Type Culture Collection (ATCC, Manassas, VA, USA). G-361 cells were cultured in McCoy’s 5A Medium (Corning, Tewksbury, MA, USA); HeLa, MCF-7,Hep-G2, RKO, FaDu, and AGS cells were cultured in Eagle’s Minimum Essential Medium (EMEM) (Corning, Tewksbury, MA, USA); and PANC-1 cells were cultured in Dulbecco’s Modified Eagle’s Medium (DMEM) (Corning, Tewksbury, MA, USA). T47-D, LNCaP cells, and A-549, NCI-H1563, and NCI-H2170 lung cancer cells were cultured in RPMI-1640 Medium (Corning, Tewksbury, MA, USA). PC-3 and DU145 cells were cultured in Kaighn’s Modification of Ham’s F-12 Medium (F12-K) (Corning, Tewksbury, MA, USA). The normal human breast epithelial cell line MCF-10 was maintained in DMEM/F12 Medium (Corning, Tewksbury, MA, USA); HUVEC cells were cultured in Endothelial Cell Growth Medium (Lonza, Basel, Switzerland); and BJ fibroblast cells were cultured in EMEM (Corning, Tewksbury, MA, USA). For all cell lines, media were supplemented with 10% fetal bovine serum (FBS) (Corning, Tewksbury, MA, USA) and cells were incubated at 37 °C in a humidified atmosphere of 5% CO_2_.

#### 4.6.2. Cytotoxic Evaluation

The MTT cytotoxicity assay was performed to assess cell viability following treatment with the tested ASEO, in accordance with a previously published protocol [41]. Cells were seeded into 96-well plates at a concentration of 2 × 10^4^ cells per well. The plates were incubated at 37 °C with 5% CO_2_ to allow for cell attachment and growth. Once the cells reached 70–80% confluence, the tested ASEO was added. The cells were treated with the tested ASEO at various concentrations ranging from 1 µg/mL to 500 µg/mL. The treatment was applied for two different incubation periods, 24 h and 48 h. After the treatment period, the MTT solution (3-(4,5-dimethylthiazol-2-yl)-2,5-diphenyltetrazolium bromide; Sigma-Aldrich, USA) was prepared at a concentration of 0.5 mg/mL. The cells were incubated with the MTT solution for 4 h at 37 °C. During this time, viable cells metabolized the MTT into formazan crystals. After the incubation period, the medium containing the MTT was carefully removed, and the formazan crystals formed by viable cells were dissolved in DMSO (POCH, Gliwice, Poland). The absorbance of the resulting solution was measured at 570 nm using a PowerWave microplate spectrophotometer (BioTek Instruments, USA). This absorbance value is proportional to the number of viable cells in each well. Cell viability was determined by comparing the absorbance of treated cells to that of untreated control cells. The IC_50_ concentration values were determined using the AAT Bioquest, Inc. Quest Graph™ IC50 Calculator [(accessed on 14 January 2020)], available online at www.aatbio.com/tools/ic50-calculator. This tool provides a statistical model for determining IC_50_ values based on the dose–response curve. Each assay was performed in triplicate, and all experiments were repeated three times to ensure the accuracy and reproducibility of the results.

#### 4.6.3. Cell Cycle Analysis

Cell cycle analysis was performed using the NucleoCounter^®^ NC-3000™ (ChemoMetec, Allerod, Denmark) in accordance with the two-step Cell Cycle Assay protocol provided by the manufacturer (application note No. 0254. Rev. 1.1, ChemoMetec, Allerod, Denmark, https://chemometec.de/wp-content/uploads/2022/08/App-note_994-0254_Two-Step-Cell-Cycle-Assay_NC-250.pdf) (accessed on 6 July 2025). T47-D cells were cultured in 6-well plates and exposed to ASEO at a concentration of their IC_50_ concentrations. Following a 48 h incubation period, the culture medium was discarded, and the cells were detached using a trypsin–EDTA solution (Corning, Tewksbury, MA, USA). The cells were then washed with PBS, resuspended in 100 μL of lysis buffer (Solution 10) containing 10 μg/mL of DAPI (4′,6-diamidine-2′-phenylindole dihydrochloride), and incubated for 5 min at 37 °C in the dark. Subsequently, 100 μL of stabilization buffer (Solution 11) was added, and 10 μL of the prepared cell suspension was applied to an NC-Slide for analysis using the NucleoCounter NC-3000.

#### 4.6.4. Analysis of Thiol Levels and Cell Vitality

The assessment of cellular thiol levels in T47D cells was performed using the NC-3000 Vitality Assay (application note No. 3005. Rev. 1.4, ChemoMetec, Allerod, Denmark, https://chemometec.fr/wp-content/uploads/2022/08/App-note_994-3005_Vitality_NC-3000.pdf) (accessed on 6 July 2025). The principle of the method is based on the reaction of the Vita Bright-48 dye with thiols and the formation of a fluorescent product. Additional dyes used in this assay included acridine orange (AO), which selectively stains dead cells, and propidium iodide (PI), which marks all nucleated cells. T47D cells were plated in 6-well plates and incubated for 48 h with ASEO at its IC_50_ concentration. Following incubation, the culture medium was removed, and the cells were detached using a trypsin–EDTA solution. Afterward, the cells were washed once with PBS and resuspended in Solution 5 (solution containing all three dyes used in the assay) at a 19:1 ratio. Subsequently, 10 µL of the cell suspension was loaded onto an NC-Slide and analyzed using the NucleoCounter NC-3000.

## 5. Conclusions

Essential oil from *A. serotina* Bunge isolated from the aerial part of native plants growing in south Kazakhstan was found to represent a new chemotype, with santolina alcohol as the main compound and the presence of other irregular monoterpenes like santolinatriene, yomogi alcohol, artemisia alcohol, lavandulol, and lavandulyl acetate. The bioactivity of this essential oil was demonstrated for the first time. These data reveal its promising antifungal and anticancer properties. Further studies are required to explore the phytochemical profiles of this endemic *Artemisia* plant in relation to its bioactivity, followed by its potential health applications. Moreover, it is also important to isolate the unidentified irregular monoterpenes and determine their structure.

## Figures and Tables

**Table 1:** Volatile compounds present in ASEO.

No.	Compound	RI_exp_	RI_lit_	Relative Percentage
1	Santolinatriene	906	909	0.2
2	Camphene	954	950	0.2
3	1-Octen-3-ol	984	962	0.2
4	Yomogi alcohol	998	991	1.1
5	[M+] 168(1), 43(100), 81(55), 95(50)	1030	-	2.1
6	Santolina alcohol	1036	1029	34.6
7	Artemisia alcohol	1084	1073	0.6
8	trans-p-Menth-2-en-1-ol	1117	1116	3.0
9	Camphor	1156	1132	7.6
10	[M+] 166(1), 79(100), 43(85), 93(80), 121(60)	1159	-	2.3
11	[M+] 152(2), 43(100), 95(55), 59(25), 138(15)	1164	-	27.0
12	Lavandulol	1169	1150	0.7
13	Borneol	1183	1159	0.3
14	Terpinen-4-ol	1189	1164	0.2
15	a-Terpineol	1204	1176	0.3
16	[M+] 168 (2), 82(100), 67(60), 110(15)	1249	-	7.1
17	Carvone	1253	1214	0.2
18	Lavandulyl acetate	1284	1275	0.2
19	(Z)-Jasmone	1401	1381	0.2
20	Germacrene D	1496	1476	0.3
21	Bicyclogermacrene	1510	1494	0.2
22	Spathulenol	1596	1572	0.7
23	Globulol	1681	1659	0.2
Total				89.5
Irregular monoterpenes				37.4
Regular monoterpenes				11.8
Sesquiterpenes				1.4
Unidentified compounds				38.5
Other				0.4

RI_exp_—retention indices on ZB-5 MS column; RI_lit_—retention indices from the literature.

**Table 2:** Antibacterial and antifungal activity of ASEO.

Bacterial Species	MIC [mg/mL]	MBC [mg/mL]	MBC/MIC	MIC for Ciprofloxacin or Vancomycin * [μg/mL]
Gram-positive bacteria				
*Staphylococcus aureus* ATCC 29213	8	16	2	0.48
*Staphylococcus aureus* ATCC 43300	8	16	2	0.24
*Staphylococcus epidermidis* ATCC 12228	8	16	2	0.12
*Enterococcus faecalis* ATCC 29212	8	16	2	0.98 *
*Micrococcus luteus* ATCC 10240	4	8	2	0.98
*Bacillus subtilis* ATCC 6633	8	16	2	0.03
*Bacillus cereus* ATCC 10876	16	16	1	0.06
Gram-negative bacteria				
*Salmonella* Typhimurium ATCC 14028	16	>16	>1	0.06
*Escherichia coli* ATCC 25922	8	16	2	0.004
*Pseudomonas aeruginosa * ATCC 27853	8	16	2	0.48
**Fungal (yeast) species**	**MIC** **[mg/mL]**	**MFC** **[mg/mL]**	**MFC/MIC**	**MIC for nystatin** **[μg/mL]**
*Candida albicans* ATCC 10231	2	4	2	0.48

The standard antimicrobial drugs used as positive controls were ciprofloxacin for bacteria (except enterococci), vancomycin * for enterococci, and nystatin (NY *) for fungi.

**Table 3:** IC_50_ values of ASEO for cancer and normal cell lines after 24 h and 48 h incubation.

Cancer Cell Line	IC_50_ [µg/mL]	IC_50_ [µg/mL]
	24 h	48 h
G-361 (*melanoma*)	273.22 ± 2.58	252.51 ± 3.45
HeLa (*cervical carcinoma*)	>500	>500
PANC-1 (*pancreatic carcinoma*)	473.21 ± 4.74	412.42 ± 4.32
MCF-7 (*breast carcinoma*)	>500	491.76 ± 2.42
T47-D (*breast carcinoma*)	40.81 ± 4.21	33.17 ± 2.11
PC-3 (*prostate carcinoma*)	>500	>500
DU-145 (*prostate carcinoma*)	>500	>500
LNCaP (*prostate carcinoma*)	>500	433.28 ± 5.63
A-549 (*lung carcinoma*)	>500	>500
NCI-H1563 (*lung carcinoma*)	258.43 ± 6.43	234.13 ± 2.86
NCI-H2170 (*lung carcinoma*)	>500	463.75 ± 4.32
Hep-G2 (*hepatocellular carcinoma*)	450.38 ± 5.21	411.23 ± 5.61
AGS (*gastric adenocarcinoma*)	471.90 ± 24.69	364.20 ± 5.89 *
FaDu (*hypopharyngeal squamous-cell carcinoma*)	393.88 ± 18.19	230.58 ± 19.01 *
RKO (*colon cancer*)	346.15 ± 29.35	161.95 ± 6.15 *
**Normal cell line**		
MCF-10 (*epithelial cells from the mammary gland*)	>500	465.62 ± 3.86
HUVEC (*endothelial cells from veins*)	>500	451.45 ± 1.78
BJ (*fibroblasts from skin*)	475.32 ± 3.86	429 ± 2.74

IC_50_ values obtained from the MTT assay were calculated using the AAT Bioquest IC50 Calculator. * after 72 h incubation.

## Data Availability

The datasets presented in this article are not readily available because the data are part of an ongoing study. Requests to access the datasets should be directed to Anna Malm, anna.malm@umlub.pl.

## Supplementary Materials

The following supporting information can be downloaded at https://www.mdpi.com/article/10.3390/molecules30142956/s1, Figure S1: Identified and unidentified monoterpenes present in ASEO; Figure S2: Antiviral activity of ASEO against CVB3. (A) CVB3-induced cytopathic effect, CVB3 virus control; (B) influence of ASEO 250 µg/mL on CVB3-infected VERO cells; influence of ribavirin (RBV) 500 mg/mL (C) or 250 mg/mL (D) on CVB3-infected VERO cells; (E) RT-qPCR amplification of RNA/cDNA of CVB3; (F) reduction in CVB3 viral load.
