# Supplementary material for: Essential Oil from the Aerial Parts of Artemisia serotina Bunge (Winter Wormwood) Growing in Kazakhstan—Phytochemical Profile and Bioactivity
Source: Molecules. 2025 Jul 14;30(14):2956. doi: 10.3390/molecules30142956 (PMC12297878; doi:10.3390/molecules30142956)
Supplement: Supplementary file 1 [file molecules-30-02956-s001.zip › molecules-3690327-supplementary.pdf]

**Supplementary Materials**

| **Identified irregular monoterpenes** | **Unidentified compounds** |
|---|---|
| 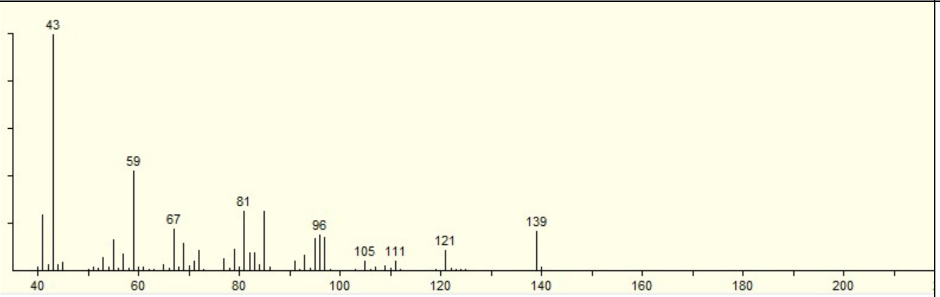 Yomogi alcohol | 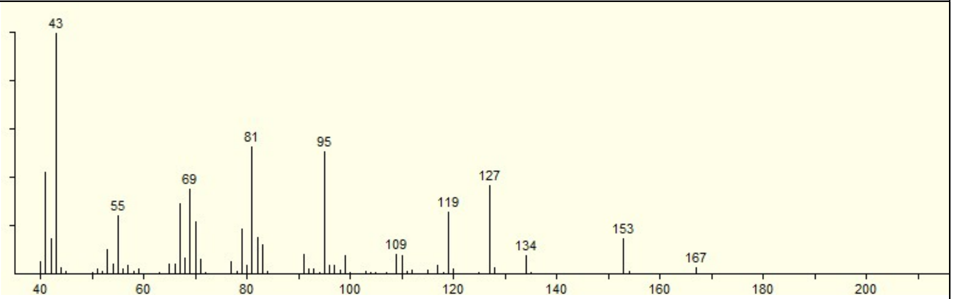 [M+] 168(1), 43(100), 81(55), 95(50) |
| 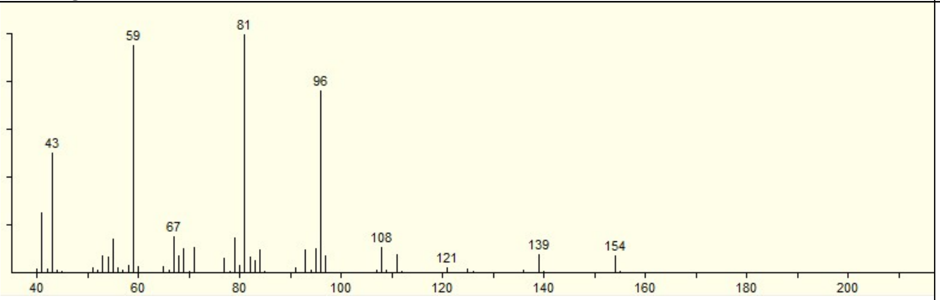 Santolina alcohol | 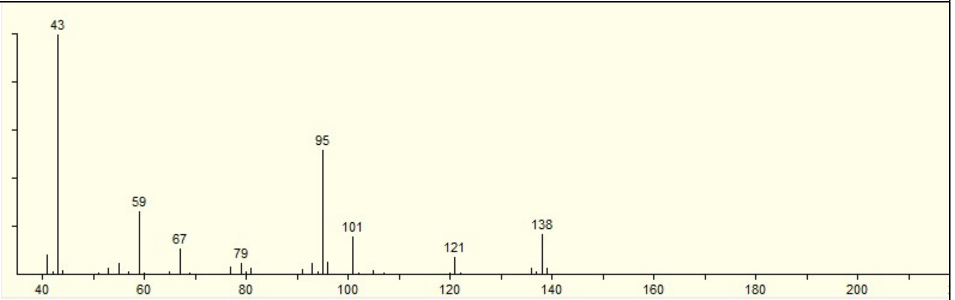 [M+] 152(2), 43(100), 95(55), 59(25), 138(15) |
| 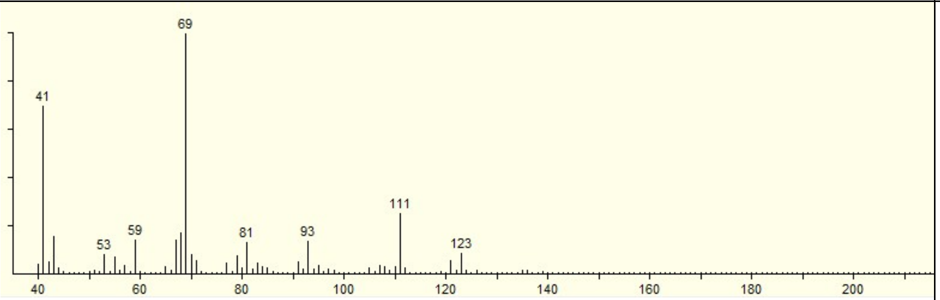 Lavandulol | 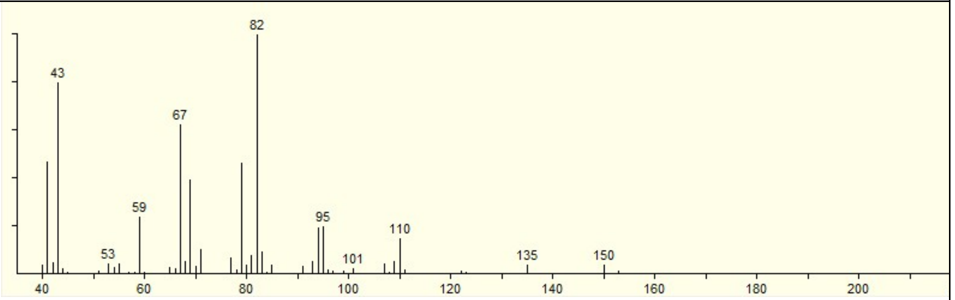 [M+] 168 (2), 82(100), 67(60), 110(15) |

Figure S1. Identified and unidentified momoterpens present in ASEO

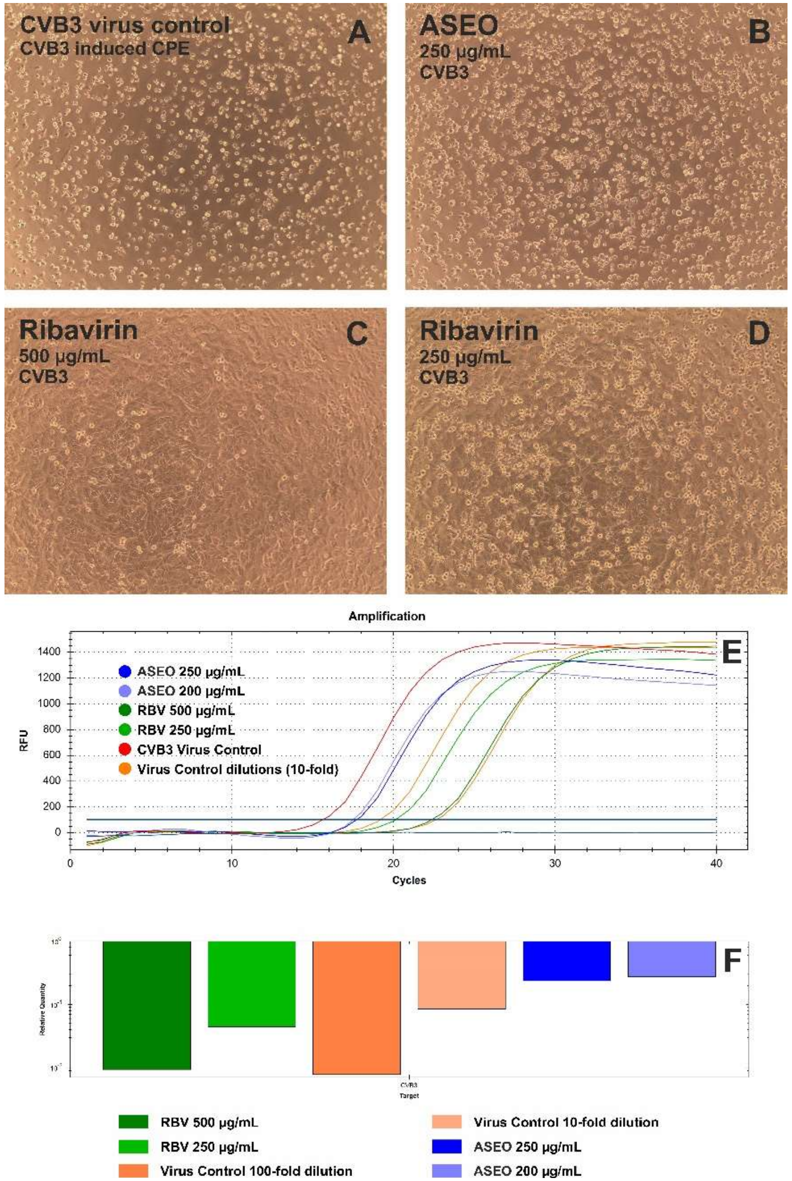
CVB3 virus control
CVB3 induced CPE
A
ASEO
250 µg/mL
CVB3
B
Ribavirin
500 µg/mL
CVB3
C
Ribavirin
250 µg/mL
CVB3
D
Amplification
E
ASEO 250 µg/mL
ASEO 200 µg/mL
RBV 500 µg/mL
RBV 250 µg/mL
CVB3 Virus Control
Virus Control dilutions (10-fold)
RFU
Cycles
F
RBV 500 µg/mL
RBV 250 µg/mL
Virus Control 100-fold dilution
Virus Control 10-fold dilution
ASEO 250 µg/mL
ASEO 200 µg/mL

Figure S2. Antiviral activity of ASEO against CVB3. (A) CVB3 induced cytopathic effect, CVB3 virus control; (B) influence of ASEO 250 µg/mL on CVB3-infected VERO cells; influence of ribavirin (RBV) 500 mg/mL (C) or 250 mg/mL (D) on CVB3-infected VERO cells; (E) RT-qPCR amplification of RNA/cDNA of CVB3; (F) reduction of CVB3 viral load.
